# Photocatalytic Optimization of ATiO_3_ Codoped with Se/Zr: A DFT Study for Hydrogen Production

**DOI:** 10.3390/ma18184389

**Published:** 2025-09-19

**Authors:** Abdellah Bouzaid, Younes Ziat, Hamza Belkhanchi

**Affiliations:** 1 Engineering and Applied Physics Team (EAPT), Superior School of Technology, Sultan Moulay Slimane University, Beni Mellal 23000, Morocco; 2 The Moroccan Association of Sciences and Techniques for Sustainable Development, Beni Mellal 23000, Morocco

**Keywords:** DFT, (Se, Zr)-codoped ATiO_3_, optical properties, photocatalytic hydrogen production

## Abstract

Recent advances in energy conversion technologies, especially solar-driven photocatalytic water splitting, are vital for satisfying the increasing global need for sustainable and clean energy. Perovskite oxides have attracted considerable attention among photocatalytic materials due to their tunable electronic structures, exceptional stability, and promise for effective hydrogen generation and environmental remediation. In this study, the optoelectronic and photocatalytic (PC) characteristics of ATiO_3_ (A = Ca, Mg) perovskites, undoped and codoped with Se and Zr, have been analyzed using ab initio simulations based on the density functional theory (DFT). The calculated formation energies for codoped systems range from −1.01 to −3.32 Ry/atom, confirming their thermodynamic stability. Furthermore, band structure calculations indicate that the undoped compounds CaTiO_3_ and MgTiO_3_ possess indirect band gaps of 2.766 eV and 2.926 eV, respectively. In contrast, codoping alters the electronic properties by changing the band gap from indirect to direct and reducing its energy, resulting in the direct band gap values 2.153 eV, 1.374 eV, 2.159 eV, and 1.726 eV for the compounds ${{Ca}_{8}Ti}_{7}{Zr}_{1}O_{23}{Se}_{1}$, ${{Ca}_{8}Ti}_{6}{Zr}_{2}O_{22}{Se}_{2}$, ${{Mg}_{8}Ti}_{7}{Zr}_{1}O_{23}{Se}_{1}$, and ${{Mg}_{8}Ti}_{6}{Zr}_{2}O_{22}{Se}_{2}$, respectively. Additionally, this codoping improves light absorption and optical conductivity in the visible and ultraviolet ranges. These enhancements become increasingly evident with elevated dopant concentrations, leading to intensified light–matter interactions. Analysis of the band edge potentials reveals that the Se-/Zr-codoped CaTiO_3_ compounds satisfy the necessary criteria for the photodissociation of water, conferring on them an ability to generate H_2_ and O_2_ under light irradiation. However, under different pH conditions, Se-/Zr-codoped MgTiO_3_ is expected to perform better at higher pH levels, while Se-/Zr-codoped CaTiO_3_ is more effective at lower pH levels. These findings highlight the promise of codoped materials for renewable energy applications, such as solar-driven hydrogen production and optoelectronic devices, with pH being a critical factor in enhancing their photocatalytic performance.

## 1. Introduction

Renewable energy solutions have emerged as a crucial global challenge amid current environmental and economic issues. Sources such as photovoltaics (PV), wind power, and batteries can satisfy rising energy demands while reducing environmental impacts [1,2,3,4]. Industrialization and economic growth have significantly increased energy consumption, depleting fossil resources and worsening pollution. Consequently, transitioning to sustainable energy sources is crucial. One promising solution is hydrogen production through water photocatalysis, which uses semiconductors to convert solar energy into clean, efficient hydrogen, providing a viable means of hydrogen generation without harming the environment [5]. Moreover, hydrogen gas (H_2_) is a clean fuel that generates no pollutants or greenhouse gases, with a high specific energy of 122 kJ/g [6,7]. Furthermore, given the pressing demand for clean and renewable sources, one of the most alluring objectives is the photocatalytic process for water splitting that is driven by solar energy.

In 1972, Fujishima and Honda commenced the investigation of TiO_2_ as a UV-responsive photocatalyst for hydrogen production [8]. Although promising, TiO_2_ exhibits a solar absorption limited to just 4% [9,10], thus restricting its efficiency. The simultaneous introduction of selenium (Se) and zirconium (Zr) as codopants modifies its electronic structure by reducing the bandgap width through the appearance of energy levels localized in the valence band (VB), thus facilitating optical transitions towards the conduction band (CB). This codoping-induced modification significantly improves the optical response of TiO_2_ and enhances its photocatalytic activity under visible light irradiation [11].

Furthermore, codoping TiO_2_ (111) with (Zr, S), (Zr, Se), and (Zr, Te) has recently enabled a decrease in the band gap, thus improving its photoactivity under visible light. Among these modifications, TiO_2_ (111) monolayers doped with tellurium (Te) and codoped (Zr, Te) have proven to be the most efficient photocatalysts for hydrogen production [12]. Furthermore, the simultaneous incorporation of selenium (Se) and nitrogen (N) into the TiO_2_ structure also leads to a reduction in the band gap, an effect attributed to the hybridization of N-2p and O-2p orbitals in the VB, as well as to the introduction of additional electronic states from Se-3p orbitals within the band gap [13].

ABX_3_ is the global chemical formula for perovskite materials, with A and B representing two cations more than B and X representing an anion. In fact, perovskite designates a range of materials that have the same atomic arrangement [14]. Moreover, perovskites are a significant material family that has properties that are suited for a variety of technological applications [15,16]. Perovskite oxides are believed to have the potential to split water and produce hydrogen through their PC activity, which is highly effective [17,18,19,20].

Numerous studies utilizing DFT have thoroughly investigated the quantum properties of perovskite compounds, particularly focusing on perovskite-based photocatalysts, especially those featuring layered or stacked structural configurations [21,22,23,24,25,26,27]. In photocatalytic water splitting, there are three main processes: On the surface of the photocatalyst, charge is transferred, solar energy is absorbed, and hydrogen is produced. For this reaction to take place, the photocatalyst must have a potential difference that is higher than the normal water potential difference of 1.23 eV [28,29,30]. The compounds CaTiO_3_ and MgTiO_3_ are noted for their photocatalytic activity, especially under UV irradiation, with band gaps of 3.4–3.51 eV [31,32] for CaTiO_3_ and 3.7–4.05 eV [33,34] for MgTiO_3_. Many oxide-based photocatalysts, including perovskite materials, often exhibit wide optical band gaps that limit their ability to absorb visible light effectively. To enhance visible light photocatalysis, researchers have explored strategies to narrow the band gap of perovskites. Among the various approaches, elemental doping has proven to be one of the most straightforward and effective methods. By introducing external dopants into the perovskite structure, it is possible to modify the electronic properties, reduce the band gap, and enhance the PC efficiency of materials like CaTiO_3_ and MgTiO_3_ within the visible spectrum [35,36,37]. Recently, La-S-doped CaTiO_3_, with a doping ratio of 0.25, demonstrates exceptional photocatalytic hydrolysis characteristics owing to its narrow band gap, rapid carrier mobility, and effective visible light absorption [37].

However, doping MgTiO_3_ with S, Se, and Te effectively reduces its band gap, enhancing visible light absorption and photocatalytic hydrogen production. Band edge analysis suggests that MgTiO_3_ doped with S (8.33–25%) and Se (8.33–16%) shows promise for solar-driven water splitting at pH = 7 due to favorable valence and conduction band positions relative to water oxidation and reduction potentials [36]. Boron (B)-doped CaTiO_3_ significantly decreases the electrical band gap, and altering the type of replacement atom can modulate the degree of this reduction, hence improving visible light absorption [38]. Previous research (Zulfiqar et al. [39]) indicates that X/Zr codoping (X = S, Se, Te) in BaTiO_3_ enhances thermodynamic stability, optical absorption, and band edge positions. Specifically, S-/Zr- and Se-/Zr-codoped BaTiO_3_ exhibited better photocatalytic hydrogen evolution performance than Te-/Zr-codoped BaTiO_3_ [39]. Moreover, (Sr, Ni) codoping in BaTiO_3_ lowers the band gap compared to pristine BaTiO_3_, and simulated optical spectroscopy indicates that the codoped materials exhibit strong dielectric and photoconductive properties [40]. Zulfiqar et al. [41] showed that codoping BaZrO_3_ with Ti and X (X = S, Se, or Te) improves the thermodynamic stability of chalcogen incorporation at oxygen sites, enabling precise bandgap tuning for efficient visible light absorption. Specifically, Zr-/Te-codoped BaZrO_3_ emerged as a promising photocatalyst for solar water splitting due to its optimal optical properties and band edge alignment for hydrogen evolution. Similarly, El Badraoui et al. [42]. Demonstrated that V and/or N doping transforms CaZrO_3_ from an indirect insulator (4.964 eV) to a direct semiconductor (1.369 eV for CaZr_0.8750_V_0.1250_O_2.9584_N_0.0416_), enhancing visible light absorption and confirming the thermodynamic stability required for solar cell applications. Moreover, Ait Brahim et al. [43] examined the impact of S, Se, and Te doping in LiNbO_3_ through the density functional theory, revealing that doping diminishes the bandgap from 3.544 eV (pure) to 2.312 eV, 1.996 eV, and 0.924 eV for S, Se, and Te doping, respectively. Te-doped LiNbO_3_ demonstrated the highest visible light absorption (exceeding 10^5^ cm^−1^) and enhanced electrical conductivity, positioning it as the most promising and environmentally sustainable candidate for solar cell applications [43]. Moreover, doping NaTaO_3_ with S, Se, or Te improves visible light photocatalysis by narrowing the bandgap and increasing light absorption. Te-doped NaTaO_3_ exhibits particularly enhanced charge separation, resulting in higher water splitting efficiency [44]. Further, Li et al. [45] synthesized S- and N-codoped NaTaO_3_ photocatalysts via a simple method. The resulting material exhibited a modified structure and enhanced visible light absorption due to the incorporation of sulfur and nitrogen.

Consequently, the catalyst degraded 95% of Rhodamine B dye in water within one hour under visible light irradiation, demonstrating high photocatalytic performance attributed to its unique structure and elemental composition. Furthermore, Lamhani et al. [46] combined experimental techniques and DFT calculations to study SrTi_1−x_Co_x_O_3−y_ (x = 0, 0.125, 0.25, 0.375, and 0.5) perovskites prepared by solid-state reaction. X-ray diffraction and Rietveld refinement confirmed that cobalt substitution maintained visible light absorption and shifted band edge positions, enhancing their suitability for water splitting. All doped samples exhibited promising PC activity for hydrogen production [46]. Further, Tareq et al. [47] established that the AlBrSe monolayer is a mechanically and thermodynamically stable indirect bandgap semiconductor, whose electronic and optical characteristics can be adeptly modified through biaxial strain, facilitating a transition to a direct bandgap and positioning it as a promising candidate for photocatalytic water splitting applications [47]. NiTiO_3_/TiO_2_ nanocomposites synthesized via a sol-gel method by Quispe Cohaila [48] exhibited enhanced photocatalytic hydrogen production. The 99.2% NiTiO_3_/0.8% TiO_2_ heterojunction significantly improved light absorption and reduced charge recombination by 85%. Incorporating TiO_2_ resulted in a decrease in crystallite size, which in turn increased the surface area and active sites, leading to a 17.1% enhancement in the hydrogen evolution rate under UV light compared to pure NiTiO_3_, demonstrating a strong potential for solar-driven renewable energy. Moreover, innovative synthesis methods, such as the mussel mimetic approach for immobilizing magnetic nanoparticles, have shown promise for pollutant removal [49], while advances in photocatalytic degradation mechanisms and membrane integration contribute to increased photocatalytic efficiency [50].

Conventional photocatalysts often exhibit wide band gaps, poor visible light absorption, misaligned band edge positions relative to the redox potentials of water, and high rates of photogenerated carrier recombination. These limitations significantly reduce their efficiency in solar-driven water splitting. This study presents a strategically designed Se/Zr codoping approach for ATiO_3_ (A = Ca, Mg) perovskites. First-principle DFT calculations demonstrate that the synergistic effects of the dopants modify the electronic structure, reduce the bandgap, improve visible light absorption, and optimally position the band edges—assessed at pH = 7—for comprehensive water splitting. Additionally, it exhibits a pH-dependent bifunctional behavior, allowing for operation as either a photocathode or a photoanode, thereby providing a versatile and efficient platform for photocatalytic applications.

## 2. Computational Methodology

The WIEN2K code (*Version 23.2* (*Release 2023*), *Vienna University of Technology*, *Austria*) [51] was used to examine perovskite structures based on DFT [52] and the Full Potential Linearized Augmented Plane Wave (FP-LAPW) method [53]. Initially, the generalized gradient approximation (GGA) was used to optimize structural parameters [54], notably the mesh volume and atomic relaxations, enabling an energetically stable basic structure to be obtained. Then, to improve the accuracy of band gap calculations, the modified Becke–Johnson potential approximation (TB-mBJ) was applied to solve the Kohn–Sham equation. This approach is known to provide bandgap values in good agreement with experimental results [55], thus correcting the underestimation typical of standard DFT methods.

Application of this methodology has enabled the structural, optoelectronic, and photocatalytic properties of ATiO_3_ (A = Ca, Mg) perovskite compounds to be studied in detail, both in the undoped and codoped with elements such as selenium (Se) and zirconium (Zr). The convergence of the wave parameters was strictly controlled to ensure the highest possible accuracy of the calculations. Specifically, we fixed the product of the muffin-tin radius (R_MT_) and the maximum wave parameter (K_MAX_) at R_MT_ × K_MAX_ = 7. This approach guarantees an accurate representation of the electronic orbitals in reciprocal space. The R_MT_ values used for each component of the materials under study are displayed in Table 1. Calculations were conducted following strict convergence criteria, established at 10^−5^ Ry for total energy and 10^−4^ e for electronic charge. Furthermore, to ensure more accuracy in sampling the reciprocal space, a thick mesh of k-points in the Brillouin zone was used, with a grid of 10 × 10 × 10 k-points. Finally, the charge localization associated with the central state was characterized by a value of −6 Rydberg, reflecting a good description of the electronic distribution and electrostatic interactions within the system.

## 3. Results and Discussions

### 3.1. Structural Properties

**Figure 1:**
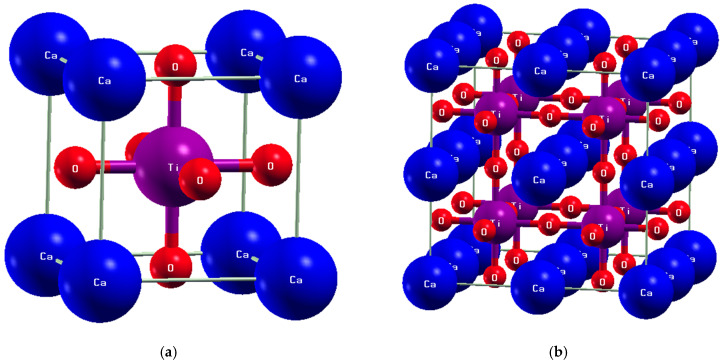
Crystal structure of ATiO_3_: (**a**) unit cell and (**b**) 2 × 2 × 2 supercell.

Perovskite-type materials have a cubic crystal structure, characterized by the Pm-3m space group (no. 221), characteristic of ATiO_3_ (A = Ca, Mg). The structure exhibits high symmetry and an ordered atomic arrangement. As shown in Figure 1a, the unit cell contains A atoms located at the corners (0, 0, 0), Ti atoms positioned at (1/2, 1/2, 1/2), and O atoms centered on the faces at (1/2, 1/2, 0). With this atomic arrangement, ATiO_3_ has high structural stability, which makes it an ideal material for numerous technological uses, particularly in optoelectronics and photocatalysis.

To study the impact of cationic and anionic substitution, a 40-atom (2 × 2 × 2) supercell of undoped cubic ATiO_3_, consisting of 8 A, 8 Ti, and 24 O atoms, was created. Two codoping strategies were then investigated to determine their effects on the material’s structural and electronic properties:

- Individual codoping: initially, Zr and Se were each introduced separately into the structure;
- Simultaneous codoping: the supercell was then simultaneously codoped with two Se and two Zr atoms to examine their combined effects on the structure and electronic characteristics.

The atomic substitution mechanism was strategically selected to ensure the charge neutrality of the system. Zr^4+^ was substituted for Ti^4+^ sites, while Se^2−^ replaced O^2−^, thus maintaining charge balance as indicated by the relationship below:

Ti^4+^ + O^2−^ = Zr^4+^ + Se^2−^

The codoped structures have chemical formulas $A_{8}{Ti}_{7}{Zr}_{1}O_{23}{Se}_{1}$ (Zr substituting for Ti and Se for O) and $A_{8}{Ti}_{6}{Zr}_{2}O_{22}{Se}_{2}$ (greater substitution of Ti by Zr and O by Se). Figure 1a,b show the optimized supercell of pure ATiO_3_ and the optimized (Se, Zr)-codoped configurations, respectively. The addition of these elements greatly alters the electronic distribution and optical properties of the compound, directly affecting its optoelectronic behavior.

The assessment of structural stability and physical properties of perovskite materials is greatly influenced by the improvement of lattice parameters and crystal volume. The performance of the material in various technological applications is influenced by key features like electron density, atomic cohesion, and interatomic interactions, which are directly linked to these parameters. A comparative analysis between experimental results and DFT theoretical data facilitated the optimization of the lattice parameter of ATiO_3_. The optimized values, presented in Table 2, demonstrate excellent agreement with experimental measurements, thereby confirming the accuracy of the theoretical model [37,56,57,58,59].

**Figure 2:**
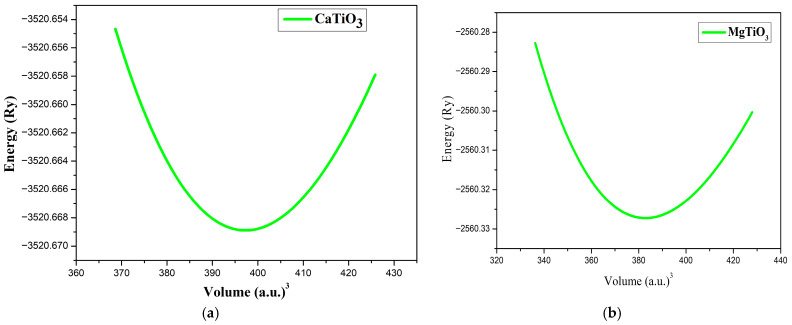
Total energy variation of (**a**) CaTiO_3_ (**b**) MgTiO_3_ as a function of volume.

To ascertain the equilibrium lattice parameters, the pressure–volume relationship of the material was modeled using the Birch–Murnaghan equation of state. This approach makes it possible to optimize the crystal volume and properly estimate the elastic properties, especially the modulus of compressibility and its pressure derivative. The Birch–Murnaghan state equation is as follows [60]:

(1) $$E_{tot}=E_{0}+\frac{9V_{0}B}{16}\left\{{\left[{\left(\frac{V_{0}}{V}\right)}^{2/3}-1\right]}^{2}B’+\left[{\left(\frac{V_{0}}{V}\right)}^{2/3}-1\right]\left[{6-4\left(\frac{V_{0}}{V}\right)}^{2/3}\right]\right\}$$

where E_tot_ is the total energy of the material, E_0_ is the ground state energy at zero pressure, V is the volume, V_0_ is the equilibrium volume, B is the bulk modulus, and B’ is the bulk modulus pressure derivative [61]. The optimization curve in Figure 2 illustrates the stability of these materials by plotting the total energy against the volume of pure ATiO_3_.

### 3.2. Formation Energy (E_f_)

The formation energies of both undoped and (Se, Zr)-codoped ATiO_3_ systems were analyzed to gain insights into their thermodynamic stability; the formula for calculating defect formation energies was applied [5,39]:

(2) $$E_{f}={(E}_{codoped}-E_{undoped})+{(n}_{Ti}\times \mu_{Ti}-n_{Zr}\times \mu_{Zr})+{(n}_{O}\times \mu_{O}-n_{Se}\times \mu_{Se})$$

where $E_{codoped}$ and $E_{undoped}$ are the total energies of the codoped and undoped supercells, respectively. The variables $\mu_{O}$, $\mu_{Ti}$, $\mu_{Se}$, and $\mu_{Zr}$ represented the chemical potentials of O, Ti, Se, and Zr, respectively, as determined by DFT calculations. Meanwhile, $n_{O}$, $n_{Ti}$, $n_{Se}$, and $n_{Zr}$ indicate the number of atoms of O, Ti, Se, and Zr that were either introduced or removed during the construction of different supercells. A negative formation energy signifies thermodynamic stability; a compound with such an energy is stable relative to its constituent elements [62,63,64]. In this study, all materials examined display this behavior, suggesting their theoretical viability and potential for experimental synthesis. Notably, undoped and codoping ATiO_3_ with (Se, Zr) results in negative formation energies, which signifies thermodynamic stability, as demonstrated in Table 3. Meanwhile, the results align with prior theoretical studies and are comparable to those of perovskites [39,57,65,66,67].

### 3.3. Electronic Properties

**Figure 3:**
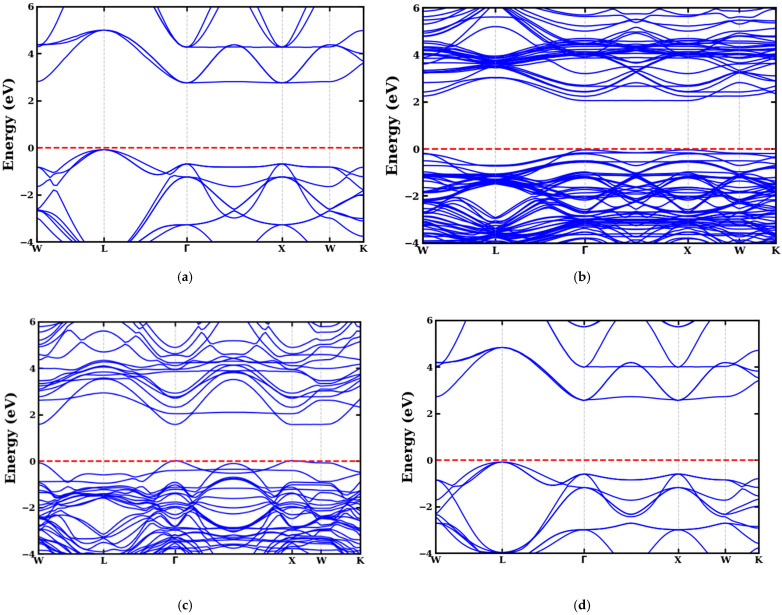
Band structure of (**a**) MgTiO_3_, (**b**) ${Mg}_{8}{Ti}_{7}{Zr}_{1}O_{23}{Se}_{1}$, (**c**) ${Mg}_{8}{Ti}_{6}{Zr}_{2}O_{22}{Se}_{2},$ and (**d**) CaTiO_3_ materials.

**Figure 4:**
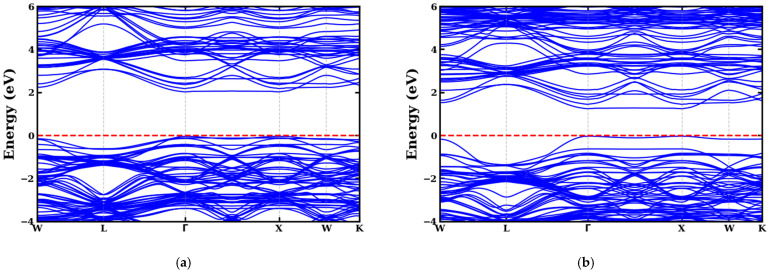
Band structure of (**a**) ${Ca}_{8}{Ti}_{7}{Zr}_{1}O_{23}{Se}_{1}$ and (**b**) ${Ca}_{8}{Ti}_{6}{Zr}_{2}O_{22}{Se}_{2}$ materials.

Analysis of the electronic structures of codoped (Se, Zr) ATiO_3_ materials is based on density of states (DOS) analysis and electronic band structures based on optimized crystal structures (Figure 1). The results obtained are illustrated in Figure 3 and Figure 4, which compare the energy bands of undoped and codoped ATiO_3_ compounds.

To accurately assess the electronic structure of the compounds, we used the mBJ approach along the high-symmetry path in the first Brillouin zone (W-L-Γ-X-W-K). This method provides a better estimate of the bandgap energy (Eg) than conventional GGA-type calculations. The results show that all undoped and codoped (Se, Zr) ATiO_3_ compounds possess a band gap, but that codoping leads to a reduction in its width (Table 4). The bandgap energy calculated for undoped ATiO_3_ compounds, where A = Ca and Mg, is 2.766 eV and 2.926 eV, respectively. These values align with prior investigations, which report values of 3.295 eV [68], 3.00 eV [56], and 3.10 eV [57], but are still lower than experimental results, which vary between 3.4 and 3.7 eV [69], with a typical value of 3.46 eV [70].

Analysis of the positions of the VBM and CBM reveals fundamental differences between undoped and codoped compounds. For undoped ATiO_3_ (A = Ca, Mg), the VBM is located at the L point and the CBM at the Γ point, indicating an indirect band gap. In contrast, for codoped ATiO_3_ (Se, Zr), both the VBM and CBM are located at the Γ point of high symmetry, reflecting a direct band gap. This transition to a direct bandgap, coupled with a reduction in bandgap energy, suggests an improvement in the optoelectronic properties of codoped materials. Thanks to these characteristics, these materials appear to be promising semiconductors for applications in photocatalysis under visible light, paving the way for advances in renewable energies and photovoltaic conversion.

Thorough analysis of the band structure is essential for understanding the effect of codoping on the electronic properties of ATiO_3_. To this end, we have studied the total density of states (TDOS) and the partial density of states (PDOS), which, respectively, examine the overall distribution of electronic states and identify the specific contributions of atomic orbitals at different energy levels.

**Figure 5:**
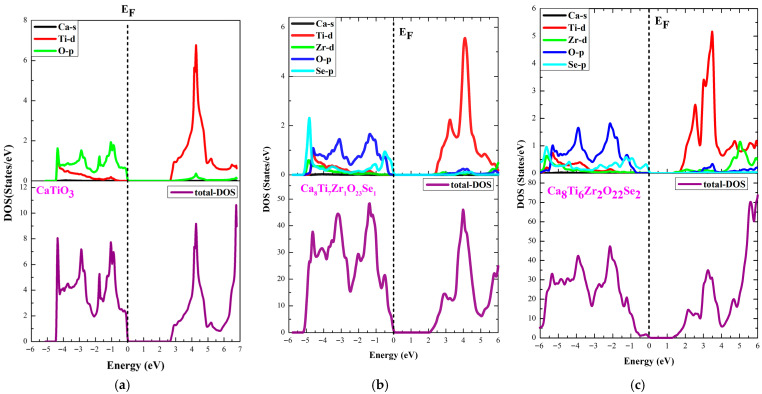
TDOS and PDOS for (**a**) CaTiO_3_, (**b**) ${Ca}_{8}{Ti}_{7}{Zr}_{1}O_{23}{Se}_{1}$, and (**c**) ${Ca}_{8}{Ti}_{6}{Zr}_{2}O_{22}{Se}_{2}$ materials.

**Figure 6:**
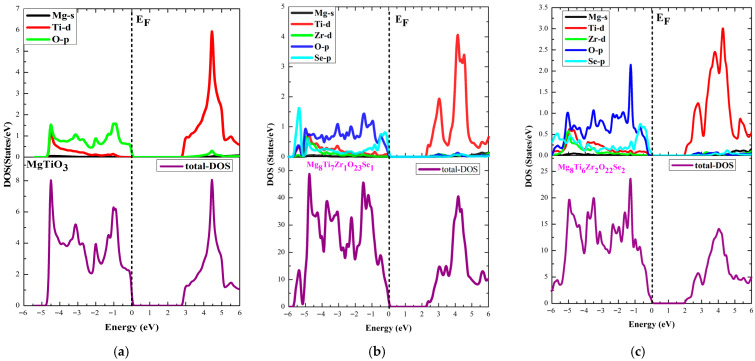
TDOS and PDOS for (**a**) MgTiO_3_, (**b**) ${Mg}_{8}{Ti}_{7}{Zr}_{1}O_{23}{Se}_{1}$, and (**c**) ${Mg}_{8}{Ti}_{6}{Zr}_{2}O_{22}{Se}_{2}$ materials.

Figure 5 and Figure 6 show the TDOS and PDOS of undoped ATiO_3_ as well as its codoped variants (Se, Zr). As illustrated in Figure 4a and Figure 5a, the electronic structure of undoped ATiO_3_ reveals that the VB, located between −6 eV and 0 eV, is predominantly composed of contributions from (Ti-d) and (O-p) orbitals. This distribution highlights strong interactions between titanium and oxygen atoms, which is characteristic of ATiO_3_-type perovskites. Furthermore, the CB, located between 0 eV and 6 eV, is dominated by (Ti-d) orbitals, suggesting that the electronic transitions responsible for conductivity originate mainly from electrons located in these orbitals. In the codoped compounds A_8_Ti_7_Zr_1_O_23_Se_1_ and A_8_Ti_6_Zr_2_O_22_Se_2_, PDOS analysis (see Figure 5 and Figure 6) highlights a redistribution of electronic states induced by the presence of Se and Zr dopants. The VB then shows a significant contribution from (Zr-d) and (Se-p) orbitals in addition to (Ti-d) and (O-p) orbitals. This modification indicates that Zr and Se atoms interact strongly with Ti and O, thus influencing the electron distribution in the VB. In the CB, although (Ti-d) orbitals remain in the majority, notable contributions from (Zr-d), (O-p), and (Se-p) orbitals are also observed. Another key point is the position of the Fermi level, which lies near the top of the VB. This feature is indicative of p-type semiconductor behavior, whatever the codoping configuration adopted. Indeed, the bandgap reduction observed in codoped systems (see Table 4) is directly linked to the introduction of dopants, which promote the formation of new electronic states near the edge of the CB. This phenomenon shifts the Fermi level upwards, improving absorption in the visible range and enhancing the material’s photocatalytic performance.

### 3.4. Optical Properties

Analysis of the absorption spectra enables precise evaluation of the optical response of the doped photocatalytic material, thus highlighting its efficiency in absorbing light [71]. In order to better understand the effect of codoping (Se, Zr) on the electronic structure of ATiO_3_, the study of optical properties is essential. This analysis relies on the calculation of the frequency-dependent dielectric function (DF), which plays a central role in the description of light–matter interactions. The complex DF of a material is represented by the subsequent expression [72,73]:

(3) $$\varepsilon (\omega )=\varepsilon_{1}(\omega )+i\varepsilon_{2}(\omega )$$

where $\varepsilon_{1}(\omega )\;$ is the real part, associated with optical dispersion, and ε_2_(ω) represents the imaginary part, which characterizes the absorption of the material at different wavelengths.

The evaluation of $\varepsilon_{2}(\omega )$ requires the study of electronic transitions between energy bands. On the other hand, the $\varepsilon_{1}(\omega )\;$ is obtained using the Kramers–Kronig transformation, which relates the dispersion and absorption of a material.

The mathematical expression of $\varepsilon_{2}(\omega )\;$ is written as follows [74,75,76]:

(4) $$\varepsilon_{2}(\omega )\;=\;(\frac{4\pi^{2}e^{2}}{m^{2}\omega^{2}})\sum_{i.j}{\left\langle i\left|M\right|j\right\rangle }^{2}f_{i}(1-f_{j})\delta (E_{f}-E_{i}-\omega )d^{3}K$$

The matrix elements governing transitions between the VB and CB are represented by M; the electron’s charge and mass are indicated by e and m, respectively; f_i_ is the Fermi distribution function for the i-th state; E_i_ is the energy of the electron in state I; and the initial and final states are denoted by i and j.

**Figure 7:**
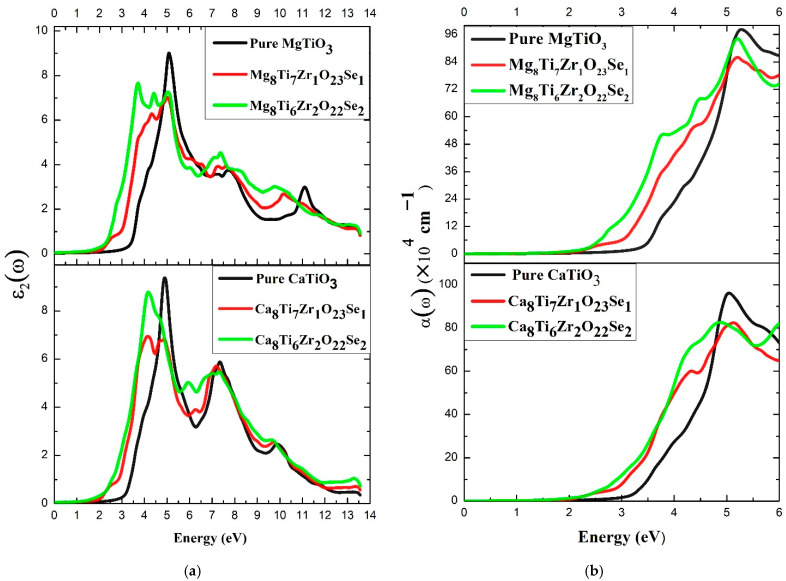
(**a**) Imaginary part and (**b**) absorption coefficient for the undoped and codoped ATiO_3_.

Figure 7a illustrates ε_2_(ω) for pure and (Se, Zr)-codoped ATiO_3_. The undoped ATiO_3_ curve (where A = Ca, Mg) shows energy thresholds near 2.77 eV and 2.93 eV, corresponding to the band gap values seen in Figure 5a and Figure 6a. Undoped electron transitions primarily lead to these thresholds in the O-2p orbital in VBM and the Ti-3d orbital in CBM. For (Se, Zr)-codoped ATiO_3_, the energy thresholds shift to lower values: 2.15 eV for Ca_8_Ti_7_Zr_1_O_23_Se_1_, 1.37 eV for Ca_8_Ti_2_Zr_2_O_22_Se_2_, 2.15 eV for Mg_8_Ti_7_Zr_1_O_23_Se_1_, and 1.81 eV for Mg_8_Ti_6_Zr_2_O_22_Se_2_. The energy gap observed in Figure 5b and Figure 6b is consistent with the decrease in onset values. Furthermore, the lower energy shifts indicate that codoping with Se and Zr enhances the visible light absorption range of ATiO_3_. This increased absorption indicates that codoping with Se and Zr significantly enhances the light absorption capacity of ATiO_3_, underscoring their potential for photovoltaic and photocatalytic applications [77]. Moreover, the results align with previous theoretical and experimental studies and are comparable to those of perovskites [5,65,71,78,79].

The following relation determines the absorption coefficient α(ω):

(5) $$\alpha (\omega )={\left(2\right)}^{1/2}\omega {\left[\sqrt{{\varepsilon_{1}(\omega )}^{2}+{\varepsilon_{2}(\omega )}^{2}}-\varepsilon_{1}(\omega )\right]}^{1/2}$$

Optical absorption of a material is initiated when the energy of an incident photon E = hν exceeds the bandgap width, allowing electrons to be excited from the VB to the CB. This process generates electron–hole pairs and triggers photovoltaic effects, an essential phenomenon in photocatalysis and solar energy conversion [68].

Figure 7b illustrates the absorption coefficient α(ω) for undoped ATiO_3_ as well as its variants codoped with Se and Zr. Pure ATiO_3_ exhibits an α(ω) in the visible range, indicating negligible absorption at these wavelengths. This low sensitivity to visible light is directly linked to its wide band gap, which limits its activation to UV wavelengths only. In contrast, codoped systems show a significant increase in α(ω), with a shift in absorption towards the visible region. This enhancement is attributed to the codoping-induced reduction in bandgap width, as confirmed in Table 4. Among the different compositions studied, Mg_8_Ti_6_Zr_2_O_22_Se_2_ shows the highest absorption in the visible region, making it more efficient than other codoped systems, including Ca_8_Ti_6_Zr_2_O_22_Se_2_, Ca_8_Ti_7_Zr_1_O_23_Se_1_, and Mg_8_Ti_7_Zr_1_O_23_Se_1_. The order of decreasing optical absorption efficiency thus follows the following trend:

Mg_8_Ti_6_Zr_2_O_22_Se_2_ > Ca_8_Ti_6_Zr_2_O_22_Se_2_ > Ca_8_Ti_7_Zr_1_O_23_Se_1_ > Mg_8_Ti_7_Zr_1_O_23_Se_1_.

This trend suggests that codoping with (Se, Zr) effectively modifies the electronic structure and widens the absorption window of the compound towards the visible spectrum. The improvement observed for Mg_8_Ti_6_Zr_2_O_22_Se_2_ could be linked to better synergy between the electronic orbitals of the dopants and those of the ATiO_3_ lattice, thus promoting more efficient electronic transitions. These findings agree with previous experimental research [80,81,82,83,84,85,86], validating the potential of Se- and Zr-codoped ATiO_3_ for photocatalytic applications. Furthermore, the results suggest that these compounds could be suitable for renewable energy applications, such as photocatalysis under solar irradiation. Additionally, they may have potential uses in photovoltaic and optoelectronic devices by utilizing visible light absorption.

The σ(ω) of a compound irradiated with photons of a specific frequency is directly influenced by the electronic conduction processes taking place within it [73]. In particular, σ(ω) is intrinsically linked to the α(ω) and to the refractive index of the material, exhibiting a similar evolution as a function of wavelength [87].

**Figure 8:**
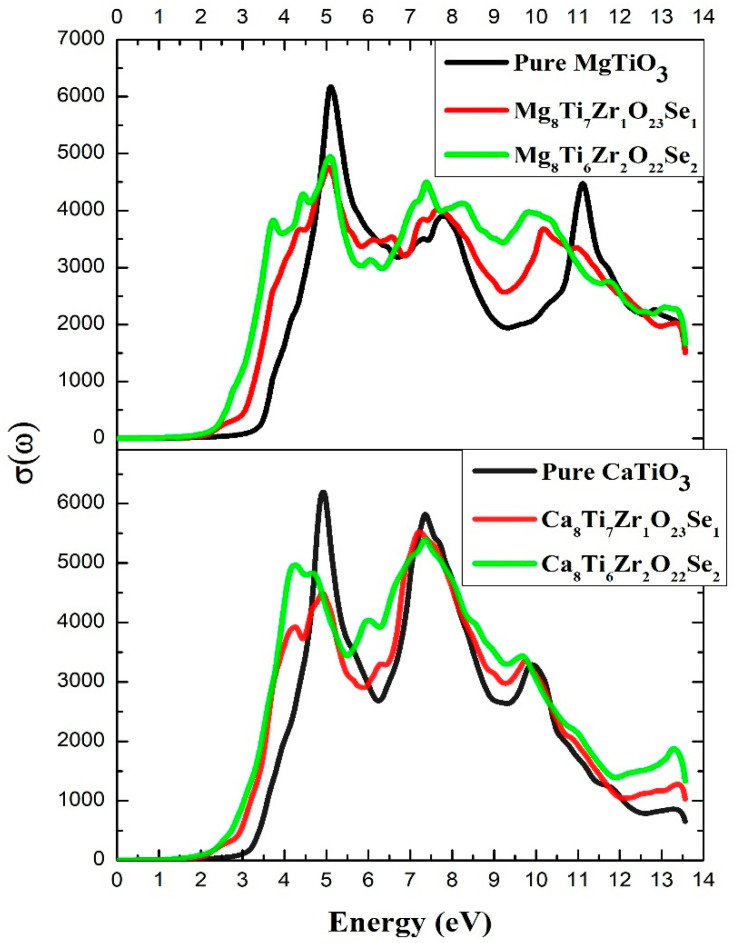
Optical conductivity of undoped and (Se, Zr)-codoped ATiO_3_.

Figure 8 illustrates σ(ω) for undoped ATiO_3_ as well as its variants codoped with Se and Zr. The results show that undoped ATiO_3_ compounds possess very low σ(ω) in the visible range, consistent with their wide bandgap limiting charge carrier excitation. However, a distinct peak appears in the ultraviolet (UV) region, indicating an efficient electronic transition at these energies. In contrast, codoped systems (Se, Zr) show a significant increase in σ(ω) in the visible spectrum, reflecting the effect of codoping on the electronic structure of the material. The reduction of the band gap explains this improvement, as it allows for the generation of charge carriers under visible light. Subsequently, the increase in σ(ω) in this range indicates a better optical response and enhanced light absorption. This indicates that these materials could be appropriate for renewable energy applications, especially in optoelectronic and photocatalytic systems that function under sunlight.

### 3.5. Energy Bands and Water Fractionation

The use of solar energy and suitable semiconductor materials to produce renewable hydrogen through photocatalytic water splitting is a promising and efficient method [88]. For optimal performance as a photocatalyst, a material must demonstrate significant absorption of solar light, especially in the visible range, and possess optimally aligned energy levels to enable the necessary redox reactions. Although the study of optical absorption properties provides useful information about the suitability of materials for various optoelectronic applications, it is insufficient in the context of photocatalytic water splitting. In this case, a thorough examination of the band edge potentials is required, as they are critical in determining the material’s ability to drive the oxidation and reduction half-reactions required for effective water dissociation.

- **Mechanism of water fractionation**

For effective photocatalytic water splitting, a material must concurrently possess an acceptable bandgap and suitably aligned band edge potentials. The bandgap energy should be between 1.23 eV and 3.0 eV to ensure sufficient solar light absorption and the energetic favorability required for water dissociation [89]. In addition to an appropriate bandgap, it is crucial to align the VBM and CBM with the water redox potentials. Specifically, the VBM must be more positive than the water oxidation potential (1.23 eV vs. NHE) to drive oxygen evolution, while the CBM must be more negative than the proton reduction potential (0 eV vs. NHE) to facilitate hydrogen generation [90,91]. Under light irradiation, the photocatalyst absorbs photons, generating electron–hole pairs. Excited electrons migrate to the CB, while holes (h^+^) remain in the VB and participate in oxidation reactions.

Charge carriers promote water dissociation reactions, which are delineated by the subsequent equations:

- Water oxidation reaction:

(6) $$H_{2}O+2h^{+}\to 2H^{+}+\frac{1}{2}O_{2}$$

- Reduction of protons to hydrogen:

**2*H*^+^ + 2*e*^−^ →*H*_2_** (7)

- Global water splitting reaction:

(8) $$H_{2}O\to H_{2}+\frac{1}{2}O_{2}$$

**Figure 9:**
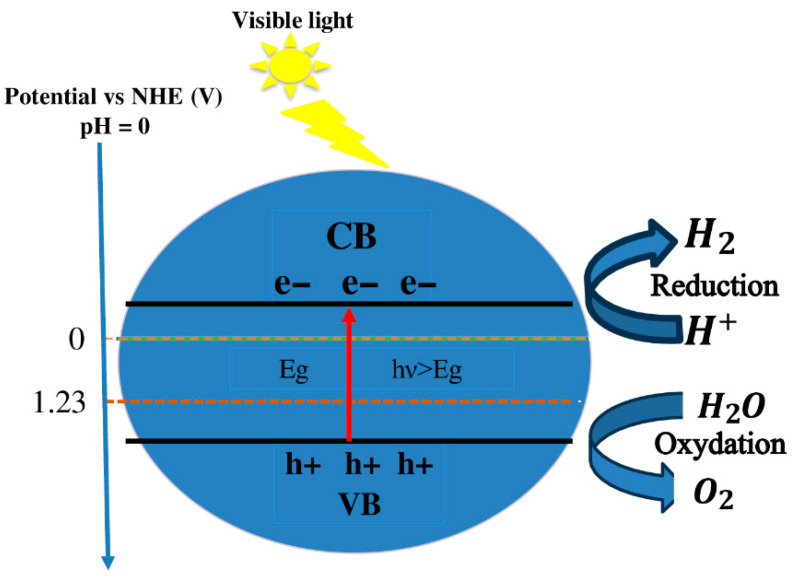
Potential photocatalytic mechanism.

In this process, electrons convert protons (H^+^) to hydrogen gas (H_2_), whereas holes oxidize water molecules to produce oxygen gas (O_2_). Optimizing the energy levels of the photocatalyst thus improves the efficiency of hydrogen production by minimizing charge recombination and maximizing sunlight absorption. The optimal reactivity for water dissociation and efficient charge separation is ensured by these conditions, as shown in Figure 9.

The electronegativity of the semiconductor (X_e_) and the Nernst equation are used to calculate the E_CB_ and E_VB_ potentials, taking care to comply with the required conditions [92,93]:

(9) $$E_{CB}=X_{e}-E_{0}-0.5E_{g}$$

(10) $$E_{VB}=X_{e}-E_{0}+0.5E_{g}$$

**Figure 10:**
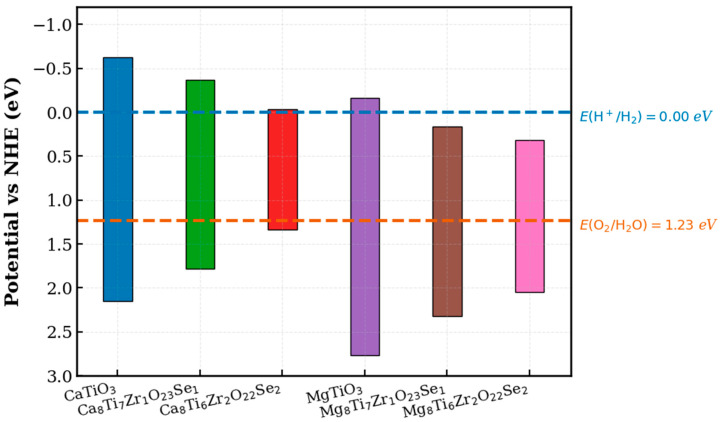
The energy band positions of both the pristine and Se-/Zr-codoped materials.

In these equations, E_0_ = 4.5 eV represents the free energy of an electron on the hydrogen scale [94], while E_VB_ and E_CB_ are the VB and CB edge potentials, respectively. Eg is the band gap energy, and X_e_ is the absolute electronegativity calculated as follows [90,93,95]:

(11) $$X_{ATiO_{3}}={\left\{\chi_{A}\times \chi_{Ti}{\times \left(\chi_{O}\right)}^{3}\right\}}^{\frac{1}{5}}$$

(12) $$X_{A_{a}{Ti}_{b}{Zr}_{c}O_{d}{Se}_{e}}={\left\{{\left(\chi_{A}\right)}^{a}\times {\left(\chi_{Ti}\right)}^{b}\times {\left(\chi_{Zr}\right)}^{c}\times {\left(\chi_{O}\right)}^{d}\times {\left(\chi_{Se}\right)}^{e}\right\}}^{\frac{1}{\left(a+b+c+d+e\right)}}$$

where $X_{ATiO_{3}}$ represent the absolute electronegativity of undoped ATiO_3_, and $X_{A_{a}{Ti}_{b}{Zr}_{c}O_{d}S_{e}}$ represents that of Se-/Zr-codoped ATiO_3_. $\chi_{A}$, $\chi_{Ti}$, $\chi_{Zr}$, $\chi_{O}$, and $\chi_{Se}$ indicate the absolute electronegativities of A, Ti, Zr, O, and Se elements, respectively, as established by Bartolotti [96]. The E_CB_ and E_VB_ potentials of (Se, Zr)-codoped ATiO_3_ were evaluated in relation to the redox potentials of water. This evaluation makes it possible to analyze their efficiency in the photodissociation of water to produce H_2_ and O_2_. Moreover, Figure 10 shows the conduction band (E_CB_) and valence band (E_VB_) edge positions for both pure and codoped compounds, offering insights into their potential photocatalytic activity. A positive VBM indicates a high oxidation capacity for holes, while a negative CBM means a high reduction potential for electrons [94,97].

These results show that the undoped ATiO_3_, ${{Ca}_{8}Ti}_{7}{Zr}_{1}O_{23}{Se}_{1}$, and ${{Ca}_{8}Ti}_{6}{Zr}_{2}O_{22}{Se}_{2}$ compounds exhibit oxidation and reduction potentials within the redox range of water, indicating their ability to produce H_2_ and O_2_ when exposed to light. The results agree well with theoretical predictions and experimental data [5,39,65,90,98,99,100,101,102,103]. For instance, compared to codoped systems such as S-/Mn-, Te-/Mn-, or Se-/Mn-codoped SrTiO_3_, X-/Zr-codoped BaTiO_3_ (X = S, Se, Te), and X-/Ti-codoped BaZrO_3_ [5,39,41,98], our Se-/Zr-codoped CaTiO_3_ exhibits more favorable band edge alignment and boosted absorption coefficients, indicating enhanced potential for solar-driven water splitting. On the other hand, the CB edge in compounds ${{Mg}_{8}Ti}_{7}{Zr}_{1}O_{23}{Se}_{1}$ and ${{Mg}_{8}Ti}_{6}{Zr}_{2}O_{22}{Se}_{2}$ is located below the water reduction potential H^+^/H_2_ (0 eV vs. NHE), which restricts the amount of H_2_ that can be produced through photocatalysis. This is because the conduction band electrons do not possess sufficient energy to reduce H^+^ ions to H_2_.

Nevertheless, the band gaps of the codoped MgTiO_3_ compounds with Se and Zr are well suited to the absorption of visible light. In summary, these values show that (Se, Zr) codoping in CaTiO_3_ aligns the bands required for the photodissociation of water, thus improving its photocatalytic reactivity for renewable energy applications. In particular, the compound ${{Ca}_{8}Ti}_{6}{Zr}_{2}O_{22}{Se}_{2}$ demonstrates a promising band gap of 1.374 eV, positioning it as a strong candidate for efficient hydrogen production from water under visible light. A comparison with previous studies indicates that Se and Zr codoping is an effective strategy for optimizing the band gap and enhancing photocatalytic performance.

### 3.6. Influence of pH on Photocatalytic Activity

**Figure 11:**
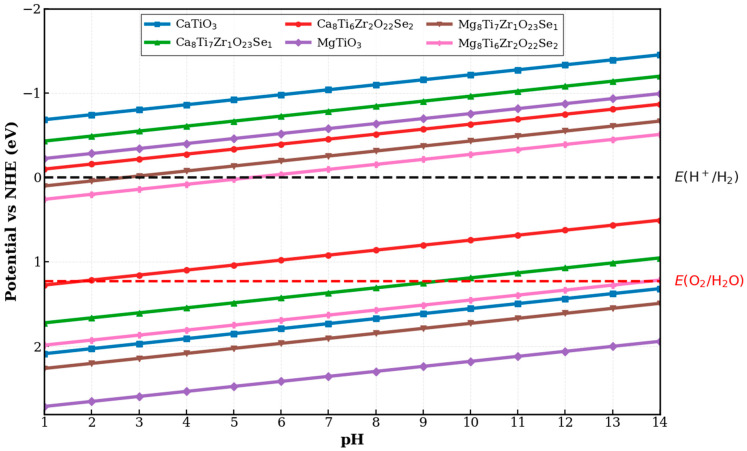
The band structure diagram shows the variation in pH-dependent band alignment for Se-/Zr-codoped ATiO_3_ (A = Ca and Mg) materials.

In the process of photocatalysis applied to water fractionation, the pH plays an essential role by influencing the redox potential, thus modifying the $E_{CB}^{pH}$ and $E_{VB}^{pH}$ energies, which are determined using the following formulas [65,93,104]:

(13) $$E_{CB}^{pH}=E_{CB}+0.05911({pH}_{pzc}-pH)$$

(14) $$E_{VB}^{pH}=E_{VB}+0.05911({pH}_{pzc}-pH)$$

where E_VB_ and E_CB_ indicate the edge potentials for the VB and CB. The photocatalyst’s ability to split water at a given pH is determined by comparing calculated band edges to pH-adjusted redox levels (CBM above H^+^/H_2_ and VBM below O_2_/H_2_O). Furthermore, studies showed that the $E_{CB}^{pH}$ values for oxide semiconductors derived from Equation (13) closely match experimental results [93]. An optimal approach for hydrogen production utilizes water as the exclusive reactant at neutral pH (about pH 7), in conjunction with an appropriate photocatalyst in direct sunlight, a process referred to as overall water splitting [105]. Figure 11 shows the energy levels of the $E_{CB}^{pH}$ and $E_{VB}^{pH}$ for (Se, Zr)-codoped ATiO_3_ at different pH levels. From Figure 11, it can be observed that across the pH range of 1 to 14, the CBM of pure ATiO_3_ (A = Ca and Mg), ${{Ca}_{8}Ti}_{7}{Zr}_{1}O_{23}{Se}_{1},$ ${{Ca}_{8}Ti}_{6}{Zr}_{2}O_{22}{Se}_{2}$, ${{Mg}_{8}Ti}_{7}{Zr}_{1}O_{23}{Se}_{1}$, and ${{Mg}_{8}Ti}_{6}{Zr}_{2}O_{22}{Se}_{2}$ lies above the water reduction potential (H^+^/H_2_) at pH ≤ 9, ≤1, >3, and >6, respectively. This indicates that under these pH conditions, these materials can effectively facilitate the reduction of water to hydrogen (H_2_), as their band edges fall within the necessary redox potential range. Consequently, they demonstrate favorable alignment for overall water splitting. However, at higher pH values for ${{Ca}_{8}Ti}_{7}{Zr}_{1}O_{23}{Se}_{1}$ and ${{Ca}_{8}Ti}_{6}{Zr}_{2}O_{22}{Se}_{2}$ (pH > 9 and pH > 1), the VBM shifts above the water oxidation potential (O_2_/H_2_O), preventing oxygen (O_2_) production through photocatalysis. In this situation, ${{Ca}_{8}Ti}_{7}{Zr}_{1}O_{23}{Se}_{1}$ and ${{Ca}_{8}Ti}_{6}{Zr}_{2}O_{22}{Se}_{2}$ function solely as photocathodes, as the photogenerated holes lack enough energy to oxidize water. Conversely, at lower pH levels for ${{Mg}_{8}Ti}_{7}{Zr}_{1}O_{23}{Se}_{1}$ and ${{Mg}_{8}Ti}_{6}{Zr}_{2}O_{22}{Se}_{2}$ (pH < 3 and pH < 6), the CBM drops below the water reduction potential, meaning the CB electrons do not have enough energy to reduce protons into hydrogen. Thus, ${{Mg}_{8}Ti}_{7}{Zr}_{1}O_{23}{Se}_{1}$ and ${{Mg}_{8}Ti}_{6}{Zr}_{2}O_{22}{Se}_{2}$ are positioned within the photoanode region under these acidic conditions and are not suitable for hydrogen production. These findings highlight the significant impact of pH on band alignment and photocatalytic efficiency. In conclusion, at pH = 7, the materials of undoped ATiO_3_, ${{Ca}_{8}Ti}_{7}{Zr}_{1}O_{23}{Se}_{1}$, ${{Mg}_{8}Ti}_{7}{Zr}_{1}O_{23}{Se}_{1}$, and ${{Mg}_{8}Ti}_{6}{Zr}_{2}O_{22}{Se}_{2}$ show appropriate band edge alignment within the water redox window, indicating their potential for photocatalytic water splitting under neutral conditions. Moreover, the results obtained agree well with other research and exhibit similar tendencies, thus affirming the dependability of the current findings and confirming the consistency of what has been observed [104,106,107,108]. Besides its photocatalytic activity for water splitting, the pH-dependent behavior of the codoped ATiO_3_ systems also indicates potential use in environmental cleanup. Under suitable pH conditions, these materials can promote the breakdown of organic pollutants, serving as effective depolluting agents. This multifunctional nature, integrating hydrogen production and pollutant removal, increases their importance for practical photocatalytic uses in both energy and environmental sectors.

## 4. Conclusions

The structural, optoelectronic, and photocatalytic characteristics of undoped ATiO_3_ compounds codoped with selenium (Se) and zirconium (Zr) were studied using DFT simulations. Electronic tape structure calculations reveal that undoped compounds, such as CaTiO_3_ and MgTiO_3_, have indirect band gaps, while codoping (Se, Zr) transforms these deviations into direct ones, thus reducing the width of the band gap and improving the absorption of visible light as well as optical conductivity. The alignment of CBM and VBM energy levels plays a crucial role in the photocatalytic activity of the materials studied. Our results show that the Se-/Zr-codoped CaTiO_3_ compounds have positions of E_VB_ and E_CB_ compatible with the redox potentials of water, indicating their capacity to produce simultaneously H_2_ and O_2_ under light irradiation. Furthermore, Se-/Zr-codoped materials, combined with an appropriate pH, are expected to be beneficial for photocatalytic applications. To conclude, this work highlights the interest of Se/Zr codoping in the optimization of material properties for water photocatalysis. It thus opens up new prospects for the development of more efficient photocatalysts suitable for applications in the field of renewable energies.

## Figures and Tables

**Table 1:** R_MT_ for the undoped and codoped ATiO_3_ compounds.

Materials	Elements	R_MT_ (a.u)
MgTiO_3_	Mg	2.5
	Ti	1.88
	O	1.70
CaTiO_3_	Ca	2.5
	Ti	1.92
	O	1.74
${{Mg}_{8}Ti}_{7}{Zr}_{1}O_{23}{Se}_{1}/{{Mg}_{8}Ti}_{6}{Zr}_{2}O_{22}{Se}_{2}$	Mg	2.42
	Ti	1.80
	Zr	1.89
	O	1.72
	Se	1.80
${{Ca}_{8}Ti}_{7}{Zr}_{1}O_{23}{Se}_{1}/{{Ca}_{8}Ti}_{6}{Zr}_{2}O_{22}{Se}_{2}$	Ca	2.45
	Ti	1.83
	Zr	1.74
	O	1.83
	Se	1.72

**Table 2:** Computed lattice parameters for ATiO_3_ compounds.

Compounds	Lattice Constant (Å)		
	Our Work	Other Study	Experimental
CaTiO_3_	3.89	3.856 [56] 3.899 [37]	3.8967 [58] 3.90 [59]
MgTiO_3_	3.8425	3.81 [57] 3.814 [56]	-

**Table 3:** Formation energy of the undoped and (Se, Zr)-codoped ATiO_3_.

Compounds	Ef (Ry/Atom)
CaTiO_3_	−0.25
MgTiO_3_	−0.24 −0.23 [57] −0.25 [66]
${Ca}_{8}{Ti}_{7}{Zr}_{1}O_{23}{Se}_{1}$	−3.32
${Ca}_{8}{Ti}_{6}{Zr}_{2}O_{22}{Se}_{2}$	−3.32
${Mg}_{8}{Ti}_{7}{Zr}_{1}O_{23}{Se}_{1}$	−2.4
${Mg}_{8}{Ti}_{6}{Zr}_{2}O_{22}{Se}_{2}$	−1.01

**Table 4:** Calculated band gap energy of pure and (Se, Zr)-codoped ATiO_3_.

Compounds	Eg (eV)
CaTiO_3_	2.766
MgTiO_3_	2.926
${Ca}_{8}{Ti}_{7}{Zr}_{1}O_{23}{Se}_{1}$	2.153
${Ca}_{8}{Ti}_{6}{Zr}_{2}O_{22}{Se}_{2}$	1.374
${Mg}_{8}{Ti}_{7}{Zr}_{1}O_{23}{Se}_{1}$	2.159
${Mg}_{8}{Ti}_{6}{Zr}_{2}O_{22}{Se}_{2}$	1.726

## Data Availability

The original contributions presented in this study are included in the article. Further inquiries can be directed to the corresponding author.
